# Training Excitatory-Inhibitory Recurrent Neural Networks for Cognitive Tasks: A Simple and Flexible Framework

**DOI:** 10.1371/journal.pcbi.1004792

**Published:** 2016-02-29

**Authors:** H. Francis Song, Guangyu R. Yang, Xiao-Jing Wang

**Affiliations:** 1 Center for Neural Science, New York University, New York, New York, United States of America; 2 NYU-ECNU Institute of Brain and Cognitive Science, NYU Shanghai, Shanghai, China; Indiana University, UNITED STATES

## Abstract

The ability to simultaneously record from large numbers of neurons in behaving animals has ushered in a new era for the study of the neural circuit mechanisms underlying cognitive functions. One promising approach to uncovering the dynamical and computational principles governing population responses is to analyze model recurrent neural networks (RNNs) that have been optimized to perform the same tasks as behaving animals. Because the optimization of network parameters specifies the desired output but not the manner in which to achieve this output, “trained” networks serve as a source of mechanistic hypotheses and a testing ground for data analyses that link neural computation to behavior. Complete access to the activity and connectivity of the circuit, and the ability to manipulate them arbitrarily, make trained networks a convenient proxy for biological circuits and a valuable platform for theoretical investigation. However, existing RNNs lack basic biological features such as the distinction between excitatory and inhibitory units (Dale’s principle), which are essential if RNNs are to provide insights into the operation of biological circuits. Moreover, trained networks can achieve the same behavioral performance but differ substantially in their structure and dynamics, highlighting the need for a simple and flexible framework for the exploratory training of RNNs. Here, we describe a framework for gradient descent-based training of excitatory-inhibitory RNNs that can incorporate a variety of biological knowledge. We provide an implementation based on the machine learning library Theano, whose automatic differentiation capabilities facilitate modifications and extensions. We validate this framework by applying it to well-known experimental paradigms such as perceptual decision-making, context-dependent integration, multisensory integration, parametric working memory, and motor sequence generation. Our results demonstrate the wide range of neural activity patterns and behavior that can be modeled, and suggest a unified setting in which diverse cognitive computations and mechanisms can be studied.

## Author Summary

Cognitive functions arise from the coordinated activity of many interconnected neurons. As neuroscientists increasingly use large datasets of simultaneously recorded neurons to study the brain, one approach that has emerged as a promising tool for interpreting population responses is to analyze model recurrent neural networks (RNNs) that have been optimized to perform the same tasks as recorded animals. Complete access to the activity and connectivity of the circuit, and the ability to manipulate them in arbitrary ways, make trained networks a convenient proxy for biological circuits and a valuable platform for theoretical investigation. However, existing RNNs lack basic biological features that are essential if RNNs are to provide insights into the circuit-level operation of the brain. Moreover, trained networks can achieve the same behavioral performance but differ substantially in their structure and dynamics, highlighting the need for a simple and flexible framework for the exploratory training of RNNs. Here we describe and provide an implementation for such a framework, which we apply to several well-known experimental paradigms that illustrate the diversity of detail that can be modeled. Our work provides a foundation for neuroscientists to harness trained RNNs in their own investigations of the neural basis of cognition.

This is a *PLOS Computational Biology* Methods paper.

## Introduction

Computations in the brain are carried out by populations of interconnected neurons. While single-neuron responses can reveal a great deal about the neural mechanisms underlying various sensory, motor, and cognitive functions, neural mechanisms often involve the coordinated activity of many neurons whose complex individual dynamics are not easily explained by tuning to experimental parameters [1–4]. A growing recognition of the importance of studying population-level responses is reflected in the increasing number of studies that use large datasets of simultaneously or sequentially recorded neurons to infer neural circuit mechanisms [5–9]. At the same time, the novel challenges posed by high-dimensional neural data have led to the development of new methods for analyzing and modeling such data [10–12].

**Fig 1:**
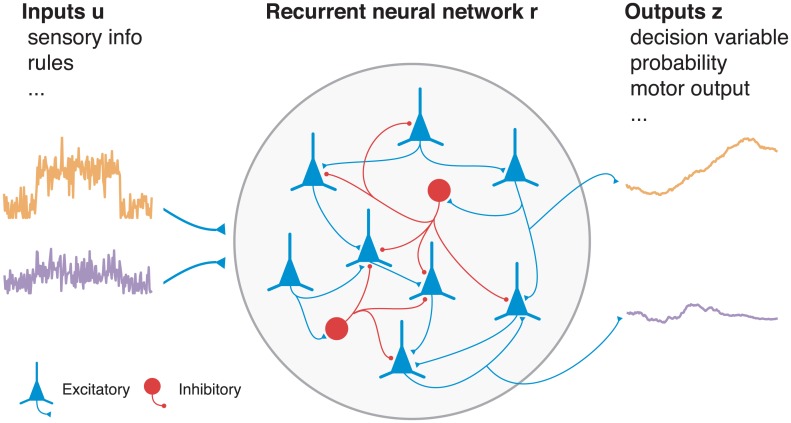
Recurrent neural network (RNN). A trained RNN of excitatory and inhibitory rate units **r**(*t*) receives time-varying inputs **u**(*t*) and produces the desired time-varying outputs **z**(*t*). Inputs encode task-relevant sensory information or internal rules, while outputs indicate a decision in the form of an abstract decision variable, probability distribution, or direct motor output. Only the recurrent units have their own dynamics: inputs are considered to be given and the outputs are read out from the recurrent units. Each unit of an RNN can be interpreted as the temporally smoothed firing rate of a single neuron or the spatial average of a group of similarly tuned neurons.

One approach that has emerged as a promising tool for identifying the dynamical and computational mechanisms embedded in large neural populations is to study model recurrent neural networks (RNNs) whose connection weights have been optimized to perform the same tasks as recorded animals [5, 7]. In [5], for example, the “trained” network was analyzed to reveal a previously unknown selection mechanism for context-dependent integration of sensory stimuli that was consistent with data obtained from behaving monkeys. RNNs of rate units, which describe biological circuits as a set of firing rates (nonlinearities) interacting through synapses (connection weights) (Fig 1), interpolate between biophysically detailed spiking-neuron models and the wider class of continuous-time dynamical systems: the units of an RNN can be interpreted as the temporal or ensemble average of one or more co-tuned spiking neurons [13], while any nonlinear dynamical system can be approximated by an RNN with a sufficient number of units [14, 15]. The optimization of network parameters typically specifies the desired output but not the manner in which to achieve this output, i.e., the *what* but not the *how*. Trained RNNs therefore serve as a source of candidate hypotheses about circuit mechanisms and a testing ground for data analyses that link neural computation to behavior. Complete access to the activity and connectivity of the circuit, and the ability to manipulate them in arbitrary ways, make trained networks a convenient proxy for biological circuits and a valuable platform for theoretical investigation [12, 16, 17].

For many tasks of interest, however, training can result in multiple networks that achieve the same behavioral performance but differ substantially in their connectivity and dynamics. As highlighted in recent work [8], the particular solution that is discovered by the training algorithm depends strongly on the set of constraints and “regularizations” used in the optimization process, so that training RNNs to perform a task is not entirely unbiased with respect to the *how*. Indeed, for the purposes of modeling animal tasks in systems neuroscience the question is no longer whether an RNN can be trained to perform the task—the answer appears to be yes in a wide range of settings—but what architectures and regularizations lead to network activity that is most similar to neural recordings obtained from behaving animals.

Answering this question is essential if RNNs are to provide insights into the operation of the brain at the level of neural circuits [18], and extends the classical connectionist approach [19, 20]. Doing so requires a simple and flexible framework for the exploratory training of RNNs to investigate the effects of different constraints on network properties, particularly those constraints that render the RNNs more biologically plausible. For instance, many RNNs studied to date have “firing rates” that are both positive and negative. More fundamentally, existing networks do not satisfy Dale’s principle [21], the basic and ubiquitous observation that neurons in the mammalian cortex have purely excitatory or inhibitory effects on other neurons. The analogous constraint that all connection weights from a given unit must have the same sign can have a profound effect on the types of dynamics, such as non-normality [22], that operate in the circuit. Moreover, connections from excitatory and inhibitory neurons exhibit different levels of sparseness and specificity, with non-random features in the distribution of connection patterns among neurons both within local circuits [23–27] and among cortical areas [28–30]. Notably, long-range projections between areas are primarily excitatory. Such details must be included in a satisfactory model of local and large-scale cortical computation.

We address this challenge by describing flexible, gradient descent-based training of excitatory-inhibitory RNNs that can incorporate a variety of biological knowledge, particularly of local and large-scale connectivity in the brain. Several different methods have previously been used to train RNNs for cognitive tasks in neuroscience, including first-order reduced and controlled error (FORCE) [7, 31, 32] and Hessian-free (HF) [5, 33, 34]. Here we use minibatch stochastic gradient descent (SGD) with the modifications described in [35], which remove the major difficulties associated with pure gradient descent training of RNNs. SGD is conceptually simple without sacrificing performance [36, 37] and is particularly advantageous in the present context for the following reasons: Unlike FORCE and like HF, SGD allows us to more easily formulate the problem of training an RNN as one of minimizing an objective function that can be modified to induce different types of solutions [8]. Meanwhile, like FORCE and unlike HF, for many tasks SGD can update parameters on a trial-by-trial basis, i.e., in an “online” fashion. This opens up the possibility of exploring across-trial effects that cannot be studied when large numbers of trials are required for each iteration of learning, as in the HF algorithm. Although none of the learning methods discussed here can at present be considered biological, recent work also suggests that spike-timing dependent plasticity (STDP) [38], which is believed to be a basic rule governing synaptic weight changes in the brain, may correspond to a form of SGD [39, 40]. However, the focus of our approach will be on the results, not the mechanism, of learning.

We provide an implementation of this framework based on the Python machine learning library Theano [41, 42], whose automatic differentiation capabilities facilitate modifications and extensions. Theano also simplifies the use of Graphics Processing Units (GPUs) when available to speed up computations. The implementation was designed to minimize the overhead for each new task by only requiring a specification of the network structure and correct input-output relationship to be learned. It also streamlines the testing and analysis of the resulting networks by using the same (customizable) specification for both training and testing (S1 Code). We demonstrate the application of this framework to well-known experimental paradigms that illustrate the diversity of tasks and details that can be modeled: perceptual decision-making, context-dependent integration, multisensory integration, parametric working memory, and eye-movement sequence generation. Using the resulting networks we perform both single-neuron and population-level analyses associated with the corresponding experimental paradigm. Our results show that trained RNNs provide a unified setting in which diverse computations and mechanisms can be studied, laying the foundation for more neuroscientists to harness trained RNNs in their own investigations of the neural basis of cognition.

## Materials and Methods

In this section we first define the RNNs used in this work, show how constraints can be introduced, then describe training the networks using a modified form of stochastic gradient descent (SGD).

### Recurrent neural networks

RNNs receive a set of *N*_in_ time-varying inputs **u**(*t*) and produce *N*_out_ outputs **z**(*t*), where inputs encode task-relevant sensory information and outputs typically represent a decision variable or probability distribution (Fig 1). Outputs can also relate to the direct motor effector, such as eye position, by which an animal indicates its decision in the behavioral paradigm. We consider RNNs whose *N* firing rates **r**(*t*) are related to their corresponding currents **x**(*t*) by the threshold (rectified) linear “*f-I* curve” [*x*]_+_ = max(*x*, 0), which maps arbitrary input currents to positive firing rates: *x* if *x* > 0 and 0 otherwise. The RNNs are described by the equations

$$\tau \dot{x}=-x+W^{\text{rec}}r+W^{\text{in}}u+\sqrt{2\tau \sigma_{\text{rec}}^{2}}\;\xi ,$$ (1)

$$r={\left[x\right]}_{+},$$ (2)

$$z=W^{\text{out}}r,$$ (3)

or, more explicitly,

$$\tau \frac{dx_{i}}{dt}=-x_{i}+\sum_{j=1}^{N}W_{ij}^{\text{rec}}r_{j}+\sum_{k=1}^{N_{\text{in}}}W_{ik}^{\text{in}}u_{k}+\sqrt{2\tau \sigma_{\text{rec}}^{2}}\;\xi_{i},$$ (4)

$$r_{i}={\left[x_{i}\right]}_{+},$$ (5)

$$z_{\ell }=\sum_{i=1}^{N}W_{\ell i}^{\text{out}}r_{i}$$ (6)

for *i* = 1, …, *N* and ℓ = 1, …, *N*_out_. In these equations *τ* is the time constant of the network units, *W*^in^ is an *N* × *N*_in_ matrix of connection weights from the inputs to network units, *W*^rec^ is an *N* × *N* matrix of recurrent connection weights between network units, *W*^out^ is an *N*_out_ × *N* matrix of connection weights from the network units to the outputs, and ***ξ*** are *N* independent Gaussian white noise processes with zero mean and unit variance that represent noise intrinsic to the network. It is worth noting that if for some ℓ = 1, …, *N*′, *N*′ ≤ *N*, the output weights $W_{\ell i}^{\text{out}}=\delta_{\ell i}$ where *δ*_*ij*_ = 1 if *i* = *j* and 0 otherwise, then the readout is the same as a subset of the network firing rates. This is useful in situations where the aim is to fix a subset of the units to experimentally recorded firing rates.

Without the rectification nonlinearity [*x*]_+_ (in which case **r** = **x**), Eqs 1–3 would describe a linear system whose dynamics is completely determined by *W*^rec^. Thus, one way to understand the effect of rectification is to consider a linear dynamical system whose coupling matrix *W*^rec^ at any given time includes only those columns that correspond to “active” units with positive summed current *x*_*i*_ (and hence positive firing rate *r*_*i*_) [43]. This toggles the network between different linear maps, thereby endowing the network with the capacity for more complex computations than would be possible with a single linear network [44, 45]. As a convenient baseline, the recurrent noise in Eq 1 has been scaled so that in the corresponding linear network without rectification each unit is an Ornstein-Uhlenbeck process with variance $\sigma_{\text{rec}}^{2}$ when *W*^rec^ = *W*^in^ = 0.

In practice, the continuous-time dynamics in Eqs 1–3 are discretized to Euler form (which we indicate by writing time as a subscript, *X*_*t*_ = *X*(*t* ⋅ Δ*t*) for a time-dependent variable *X*) in time steps of size Δ*t* as [46]

$$x_{t}=\left(1-\alpha \right)x_{t-1}+\alpha \left(W^{\text{rec}}r_{t-1}+W^{\text{in}}u_{t}\right)+\sqrt{2\alpha \sigma_{\text{rec}}^{2}}\;N\left(0,1\right),$$ (7)

$$r_{t}={\left[x_{t}\right]}_{+},$$ (8)

$$z_{t}=W^{\text{out}}r_{t},$$ (9)

where *α* = Δ*t*/*τ* and **N**(0, 1) are normally distributed random numbers with zero mean and unit variance, sampled independently at every time step. In this formulation, the usual discrete-time RNNs used in machine learning applications correspond to *α* = 1 or Δ*t* = *τ*. To minimize computational effort we train the network with a value of Δ*t* that is as large as possible such that the same network behavior is recovered in the continuous limit of Δ*t* → 0.

Although the details of the inputs to the network are specific to each task, it is convenient to represent all inputs as a rectified sum of baseline **u**^0^, task-dependent signal **u**^task^(*t*), and Gaussian white noise *ξ*:

$$u\left(t\right)={\left[u^{0}+u^{\text{task}}\left(t\right)+\sqrt{2\tau \sigma_{\text{in}}^{2}}\;\xi \right]}_{+}$$ (10)

in the continuous description, and

$$u_{t}={\left[u^{0}+u_{t}^{\text{task}}+\frac{1}{\alpha }\sqrt{2\alpha \sigma_{\text{in}}^{2}}\;N\left(0,1\right)\right]}_{+}$$ (11)

in the discrete-time description. Motivated by the interpretation that the network under study is only one part of a larger circuit, the baseline and noise terms in the inputs can together be considered the spontaneous firing rate of “upstream” units that project to the network.

We note that in Eq 1 the external “sensory” noise ultimately combines with the intrinsic noise, with the difference that input noise can be shared between many units in the network while the recurrent noise is private to each unit. There are many cases where the external and internal noise trade off in their effect on the network, for instance on its psychometric performance in a perceptual decision-making task. However, the two sources of noise can be biologically and conceptually quite different [47], and for this reason it is helpful to separate the two types of noise in our formulation.

Finally, in many cases (the exception being networks that are run continuously without reset) it is convenient to optimize the initial condition **x**_0_ = **x**(0) at time *t* = 0 along with the network weights. This merely selects a suitable starting point for each run, reducing the time it takes for the network to relax to its spontaneous state in the absence of inputs. It has little effect on the robustness of the network due to the recurrent noise used both during and after training; in particular, the network state at the time of stimulus onset is highly variable across trials.

### RNNs with separate excitatory and inhibitory populations

A basic and ubiquitous observation in the mammalian cortex, known in the more general case as Dale’s principle [21], is that cortical neurons have either purely excitatory or inhibitory effects on postsynaptic neurons. Moreover, excitatory neurons outnumber inhibitory neurons by a ratio of roughly 4 to 1. In a rate model with positive firing rates such as the one given by Eqs 1–3, a connection from unit *j* to unit *i* is “excitatory” if $W_{ij}^{\text{rec}}>0$ and “inhibitory” if $W_{ij}^{\text{rec}}<0$. A *unit* *j* is excitatory if all of its projections on other units are zero or excitatory, i.e., if $W_{ij}^{\text{rec}}\geq 0$ for all *i*; similarly, unit *j* is inhibitory if $W_{ij}^{\text{rec}}\leq 0$ for all *i*. In the case where the outputs are considered to be units in a downstream network, consistency requires that for all ℓ the readout weights satisfy $W_{\ell j}^{\text{out}}\geq 0$ and $W_{\ell j}^{\text{out}}\leq 0$ for excitatory and inhibitory units *j*, respectively. Since long-range projections in the mammalian cortex are exclusively excitatory, for most networks we limit readout to the excitatory units. It is also natural in most cases to assume that inputs to the network are long-range inputs from an upstream circuit, and we assume all elements of the input weight matrix *W*^in^ are non-negative. For consistency with the following, we indicate this as *W*^in^ = *W*^in,+^. Once again, this is only meaningful if the inputs themselves are always non-negative, motivating the rectification of inputs in Eq 10.

In order to train RNNs that satisfy the above constraints, we parametrize the recurrent weight matrix *W*^rec^ as the product of a non-negative matrix *W*^rec,+^ and a diagonal matrix *D* of 1’s and −1’s, *W*^rec^ = *W*^rec,+^ *D*. For example, consider a network containing 4 excitatory units and 1 inhibitory unit; the excitatory/inhibitory signature of the network is then *D* = *diag*(1, 1, 1, 1, −1) (a matrix with the specified entries on the diagonal and zeros everywhere else), and the full recurrent weight matrix has the form

$$\underset{W^{\text{rec}}}{\underset{︸}{\left(\begin{array}{ccccc} & + & + & + & -\\ + & & + & + & -\\ + & + & & + & -\\ + & + & + & & -\\ + & + & + & + \end{array}\right)}}=\underset{W^{\text{rec,+}}}{\underset{︸}{\left(\begin{array}{ccccc} & + & + & + & +\\ + & & + & + & +\\ + & + & & + & +\\ + & + & + & & +\\ + & + & + & + \end{array}\right)}}\underset{D}{\underset{︸}{\left(\begin{array}{ccccc} 1 & & & & \\ & 1 & & & \\ & & 1 & & \\ & & & 1 & \\ & & & & -1 \end{array}\right)}},$$ (12)

where absent matrix elements indicate zeros. Although an individual unit in an RNN does not necessarily represent a single neuron, we typically fix the self-connections represented by the diagonal elements of *W*^rec^ to be zero, see below. Similarly, if the readout from the network is considered to be long-range projections to a downstream network, then the output weights are parametrized as *W*^out^ = *W*^out,+^ *D*.

During training, the positivity of *W*^in,+^, *W*^rec,+^, and *W*^out,+^ can be enforced in several ways, including rectification [*W*]_+_ and the absolute value function |*W*|. Here we use rectification.

### Specifying the pattern of connectivity

In addition to dividing units into separate excitatory and inhibitory populations, we can also constrain their pattern of connectivity. This can range from simple constraints such as the absence of self-connections to more complex structures derived from biology. Local cortical circuits have distance [48], layer [26, 49, 50], and cell-type [23, 25, 27, 51] dependent patterns of connectivity and different overall levels of sparseness for excitatory to excitatory, inhibitory to excitatory, excitatory to inhibitory, and inhibitory to inhibitory connections [52, 53]. Although the density of connections in a trained network can be either fixed (hard constraint) or induced through regularization (soft constraint) (see Eq 27), here we focus on the former to address the more general problem of imposing known biological structure on trained networks. For instance, in models of large-scale, distributed computation in the brain we can consider multiple cortical “areas” characterized by local inhibition within areas and long-range excitation between areas. These long-range connections can be distributed according to a highly complex topology [28–30]. It is also desirable when testing specific hypotheses about circuit structure to fix a subset of the connection weights to predefined values while leaving others as “plastic,” modifiable by training.

A simple way to impose hard constraints on the connectivity is to parametrize the weight matrices using masks. As an example, suppose we would like to train a subset of the excitatory weights and also fix two of the inhibitory weights to *w*_1_ and *w*_2_ so that they are not modified during training. We can implement this by writing

$$W^{\text{rec,+}}=\underset{M^{\text{rec}}}{\underset{︸}{\left(\begin{array}{ccccc} 0 & 1 & 1 & 1 & 0\\ 1 & 0 & 1 & 0 & 1\\ 1 & 1 & 0 & 1 & 1\\ 0 & 1 & 1 & 0 & 0\\ 1 & 1 & 1 & 1 & 0 \end{array}\right)}}⊙\underset{W^{\text{rec,plastic,+}}}{\underset{︸}{\left(\begin{array}{ccccc} \cdot & + & + & + & \cdot \\ + & \cdot & + & \cdot & +\\ + & + & \cdot & + & +\\ \cdot & + & + & \cdot & \cdot \\ + & + & + & + & \cdot \end{array}\right)}}+\underset{W^{\text{rec,fixed,+}}}{\underset{︸}{\left(\begin{array}{ccccc} 0 & 0 & 0 & 0 & w_{1}\\ 0 & 0 & 0 & 0 & 0\\ 0 & 0 & 0 & 0 & 0\\ 0 & 0 & 0 & 0 & w_{2}\\ 0 & 0 & 0 & 0 & 0 \end{array}\right)}},$$ (13)

where ⊙ denotes the element-wise multiplication of two matrices (not standard matrix multiplication). Here *W*^rec,plastic,+^ is obtained by rectifying the (unconstrained) trained weights *W*^rec,plastic^, so that *W*^rec,plastic,+^ = [*W*^rec,plastic^]_+_, while *W*^rec,fixed,+^ is a matrix of fixed weights. The elements that are marked with a dot are irrelevant and play no role in the network’s dynamics. Eq 13 has the effect of optimizing only those elements which are nonzero in the multiplying mask *M*^rec^, which ensures that the weights corresponding to zeros do not contribute. Some elements, for instance the inhibitory weights *w*_1_ and *w*_2_ in Eq 13, remain fixed at their specified values throughout training. Explicitly, the full weight matrix of the RNN is related to the underlying trained weight matrix *W*^rec,plastic^ by (cf. Eq 12)

$$W^{\text{rec}}=\left(M^{\text{rec}}⊙{\left[W^{\text{rec,plastic}}\right]}_{+}+W^{\text{rec,fixed,+}}\right)D,$$ (14)

and similarly for the input and output weights.

### Initialization

In networks that do not contain separate excitatory and inhibitory populations, it is convenient to initialize the recurrent weight matrix as $W^{\text{rec}}=\rho W_{0}^{\text{rec}}$, where $W_{0}^{\text{rec}}$ is formed by setting a fraction *p*, 0 < *p* ≤ 1, of elements to nonzero values drawn from a Gaussian distribution with mean 0 and variance (*pN*)^−1^, and the remaining fraction 1 − *p* to zero [31]. This can be understood as first generating a random matrix $W_{0}^{\text{rec}}$, then multiplying by *ρ*/*ρ*_0_ where *ρ*_0_ = 1 is the spectral radius of $W_{0}^{\text{rec}}$ and *ρ* is the desired spectral radius of the initial weight matrix. Here the spectral radius is the largest absolute value of the eigenvalues.

To initialize an excitatory-inhibitory network with an arbitrary pattern of connections, we similarly first generate a matrix $W_{0}^{\text{rec}}$ and let $W^{\text{rec}}=\left(\rho /\rho_{0}\right)W_{0}^{\text{rec}}$ where *ρ*_0_ is the spectral radius of $W_{0}^{\text{rec}}$. Unlike in the case of random Gaussian matrices, the (asymptotically) exact spectral radius is usually unknown and must be computed numerically. Moreover, since the signs of the matrix elements are determined by the excitatory or inhibitory nature of the units, it is more natural to use a distribution over positive numbers to first generate $W_{0}^{\text{rec,+}}$ (Eq 12). Many distributions, including the uniform and log-normal distributions, can be used; inspired by previous work [54], here we use the gamma distribution to initialize the recurrent weight matrix $W_{0}^{\text{rec,+}}$. The means *μ*_*E*_ (excitatory) and *μ*_*I*_ (inhibitory) of the gamma distributions are chosen to balance the excitatory and inhibitory inputs to each unit [55], i.e., ∑_*j* ∈ exc_ |*μ*_*j*_| = ∑_*j* ∈ inh_ |*μ*_*j*_|, with the overall mean set by the imposed spectral radius *ρ*. We did not use the “initialization trick” of [56], as this requires the existence of self-connections.

For the input weight matrix $W_{0}^{\text{in,+}}$ and output weight matrix $W_{0}^{\text{out,+}}$, we initialize with small positive numbers drawn from a uniform distribution.

### Training RNNs with gradient descent

To train an RNN, we assume that at each time step (or subset of time steps) there is a correct set of target outputs $z_{t}^{\text{target}}$ that depend on the current and previous history of inputs **u**_*t*′_ for *t*′ ≤ *t*, i.e., we only consider tasks that can be translated into a “supervised” form. The goal is then to find network parameters, which we collectively denote as ***θ***, that minimize the difference between the correct output and the actual output of the network. More generally, we minimize an objective function E(θ) that includes not only this error but other terms such as an *L*_1_-regularization term (for encouraging sparse weights or activation patterns) that influence the types of solutions found by the training algorithm. We begin with the case where the objective function depends only on the error; one possibility for the *loss* L(θ) that measures the difference between the correct and actual outputs is the squared sum of differences averaged over *N*_trials_ trials, *N*_out_ outputs, and *N*_time_ time points:

$$E=\frac{1}{N_{\text{trials}}}\sum_{n=1}^{N_{\text{trials}}}L_{n},$$ (15)

$$L_{n}=\frac{1}{N_{\text{out}}N_{\text{time}}}\sum_{\ell =1}^{N_{\text{out}}}\sum_{t=1}^{N_{\text{time}}}M_{t\ell }^{\text{error}}{\left[{\left(z_{t}\right)}_{\ell }-{\left(z_{t}^{\text{target}}\right)}_{\ell }\right]}^{2}.$$ (16)

For each trial *n* in Eq 16, (**z**_*t*_)_ℓ_ is the ℓ-th output, at time *t*, of the discretized network in Eq 9. The error mask *M*^error^ is a matrix of ones and zeros that determines whether the error in output ℓ at time *t* should be taken into account. In many decision-making tasks, for example, this allows us to train networks by specifying only the final, but not the intermediate, time course for the outputs.

In gradient descent training the parameters of the network are updated iteratively according to (for more sophisticated forms of gradient descent see, e.g., [57])

$$\theta^{\left(i\right)}=\theta^{\left(i-1\right)}+\delta \theta^{\left(i-1\right)},$$ (17)

where *i* denotes the iteration. The parameter change, ***δθ***, is taken to be proportional to the negative gradient of the objective function with respect to the network parameters as

$$\delta \theta^{\left(i-1\right)}=-\eta \nabla E^{\left(i-1\right)},$$ (18)

where *η* is the *learning rate* and $\nabla E^{\left(i-1\right)}=\nabla E\left(\theta^{\left(i-1\right)}\right)$ is the value of the gradient evaluated on the parameters from iteration *i* − 1. Importantly, the required gradient can be computed efficiently by backpropagation through time (BPTT) [58] and *automatically* by the Python machine library Theano [41, 42]. In component form the parameter update at iteration *i* is given by

$$\theta_{k}^{\left(i\right)}=\theta_{k}^{\left(i-1\right)}-\eta {\left(\frac{\partial E}{\partial \theta_{k}}\right)}^{\left(i-1\right)},$$ (19)

where *k* runs over all the parameters of the network that are being optimized. Eqs 17 and 18 are motivated by the observation that, for a small change ***δθ*** in the value of the parameters, the corresponding change in the value of the objective function is given by

E(θ+δθ)-E(θ)≃∇E·δθ=|∇E||δθ|cosϕ, (20)

where |⋅| denotes the norm of a vector and *ϕ* is the angle between ∇E and ***δθ***. This change is most negative when *ϕ* = 180°, i.e., when the change in parameters is in the opposite direction of the gradient. “Minibatch stochastic” refers to the fact that the gradient of the objective function E(θ) is only *approximated* by evaluating E(θ) over a relatively small number of trials (in particular, smaller than or comparable to the number of trial conditions) rather than using many trials to obtain the “true” gradient. Intuitively, this improves convergence to a satisfactory solution when the objective function is a highly complicated function of the parameters by stochastically sampling the gradient and thereby escaping saddle points [59] or poor local minima, while still performing an averaged form of gradient descent over many stochastic updates.

Even so, SGD with the objective function given in Eqs 15 and 16 often fails to converge to a solution when the network must learn dependencies between distant time points [60]. To remedy this problem, which is due to some gradient components being too large (*exploding* gradients) and some gradient components being too small (*vanishing* gradients), we follow [35] in making two modifications. First, the exploding gradient problem is addressed by simply “clipping” the gradient when its norm exceeds a maximum *G*: instead of Eq 18 for the direction and size of the update, we use

$$\delta \theta^{\left(i-1\right)}=\{\begin{array}{ll} -\eta \nabla {ℰ}^{\left(i-1\right)}\times \frac{G}{\left|\nabla {ℰ}^{\left(i-1\right)}\right|} & \text{if}\;|\nabla {ℰ}^{\left(\text{i-1}\right)}|>\text{G},\\ -\eta \nabla {ℰ}^{\left(i-1\right)} & \text{otherwise}. \end{array}$$ (21)

Second, the vanishing gradient problem is addressed by modifying the objective function with the addition of a regularization term:

$$E=\frac{1}{N_{\text{trials}}}\sum_{n=1}^{N_{\text{trials}}}\left(L_{n}+\lambda_{\Omega }\Omega_{n}\right),$$ (22)

$$\Omega_{n}=\sum_{t=1}^{N_{\text{time}}}{\left(\frac{|\frac{\partial L_{n}}{\partial x_{t}}\frac{\partial x_{t}}{\partial x_{t-1}}{|}^{2}}{|\frac{\partial L_{n}}{\partial x_{t}}{|}^{2}}-1\right)}^{2}.$$ (23)

In Eq 22 the multiplier *λ*_*Ω*_ determines the effect of the regularization term *Ω*_*n*_, with no effect for *λ*_*Ω*_ = 0. In Eq 23, the first term in parentheses is the ratio between the squared norms of two vectors, which we would like to be close to 1. The somewhat opaque (row) vector expression in the numerator can be unpacked as (cf. Eq 7)

$${\left(\frac{\partial L_{n}}{\partial x_{t}}\frac{\partial x_{t}}{\partial x_{t-1}}\right)}_{j}=\sum_{k=1}^{N}\frac{\partial L_{n}}{\partial {\left(x_{t}\right)}_{k}}\frac{\partial {\left(x_{t}\right)}_{k}}{\partial {\left(x_{t-1}\right)}_{j}}$$ (24)

$$={\left[\left(1-\alpha \right)\frac{\partial L_{n}}{\partial x_{t}}+\alpha \left(\frac{\partial L_{n}}{\partial x_{t}}W^{\text{rec}}\right)⊙\left(r_{t-1}^{\prime }\right)\right]}_{j}.$$ (25)

Here each component *r*′(*x*_*t*_) of **r**′(**x**_*t*_) is the derivative of the *f-I* curve, i.e., 1 if *x* > 0 and 0 otherwise in the case of rectification, and ⊙ denotes element-wise multiplication of two vectors. For consistency in notation we treat $r_{t-1}^{\prime }$ here as a row vector. One subtlety in the implementation of this term is that, for computational efficiency, only the “immediate” derivative of *Ω*_*n*_ with respect to the network parameters is used, i.e., with **x**_*t*_ and $\partial L_{n}/\partial x_{t}$ treated as constant [35]. The relevant network parameters in this case are the elements of the trained weight matrix *W*^rec,plastic^, which is related to *W*^rec^ through Eq 14.

The role of the regularization term *Ω*_*n*_ is to preserve the size of the gradients as errors are backpropagated through time. This is accomplished by preserving the norm of ∂**x**_*t*_/∂**x**_*t*−1_, which propagates errors in time [35], along $\partial L_{n}/\partial x_{t}$, which is the direction in which the change in the objective function is greatest with respect to **x**_*t*_. More intuitively, the impact of the regularization term on network dynamics can be understood by noting that if ∂**x**_*t*_/∂**x**_*t*′_ is small for some *t*′ < *t* then, by definition, **x**_*t*_ does not depend on small changes in **x**_*t*′_, which may occur when **x** is close to an attractor. Preserving the norm of ∂**x**_*t*_/∂**x**_*t*−1_ through time therefore encourages the network to remain at the boundaries between basins of attraction and thus encourages longer computation times. For instance, this results in perceptual decision networks that can integrate their inputs for a long period of time, before converging to one of the choice attractors. We note that, although the numerator and denominator in Eq 23 appear, by the chain rule, to preserve the ratio of $\partial L_{n}/\partial x_{t-1}$ to $\partial L_{n}/\partial x_{t}$, this is only approximately true. Specifically,

$$\frac{\partial L_{n}}{\partial x_{t}}\frac{\partial x_{t}}{\partial x_{t-1}}=\frac{\partial L_{n}}{\partial x_{t-1}}-\frac{\partial L_{n,t-1}}{\partial x_{t-1}},$$ (26)

because $L_{n,t-1}$, the component of $L_{n}$ from time *t* − 1, depends on **x**_*t*−1_ but not on **x**_*t*_.

Finally, additional regularization terms may be included to change either the dynamics or the connectivity. For instance, there are two ways of obtaining sparse recurrent connectivity. First, we can impose a hard constraint that fixes a chosen subset of weights to be nonzero and modifiable by the optimization algorithm as described above. Second, we may apply a soft constraint by adding the sum of the *L*_1_-norms of the weights to the objective function:

$$E=\frac{1}{N_{\text{trials}}}\sum_{n=1}^{N_{\text{trials}}}\left(L_{n}+\lambda_{\Omega }\Omega_{n}\right)+\frac{\lambda_{1}^{\text{rec}}}{N^{2}}\sum_{j,k=1}^{N}\left|W_{jk}^{\text{rec}}\right|.$$ (27)

In addition, we may choose to encourage solutions with small firing rates through regularization of the *L*_2_-norms of the firing rates [8]:

$$E=\frac{1}{N_{\text{trials}}}\sum_{n=1}^{N_{\text{trials}}}\left(L_{n}+\lambda_{\Omega }\Omega_{n}+\lambda_{2}^{\text{fr}}R_{n}^{\text{fr}}\right)+\frac{\lambda_{1}^{\text{rec}}}{N^{2}}\sum_{j,k=1}^{N}\left|W_{jk}^{\text{rec}}\right|,$$ (28)

$$R_{n}^{\text{fr}}=\frac{1}{NN_{\text{time}}}\sum_{j=1}^{N}\sum_{t=1}^{N_{\text{time}}}{\left(r_{t}\right)}_{j}^{2}$$ (29)

where (**r**_*t*_)_*j*_ is the firing rate of the *j*-th unit at time *t* on each trial. Again, we gain flexibility in defining more complex regularization terms because Theano computes the necessary gradients using BPTT. Although BPTT is simply a specialized chain rule for neural networks, automatic differentiation frees us from implementing new gradients each time the objective function is changed. This greatly facilitates the exploration of soft constraints such as those considered in [8].

### Training protocol

To demonstrate the robustness of the training method, we used many of the same parameters to train all tasks (Table 1). In particular, the learning rate *η*, maximum gradient norm *G*, and the strength *λ*_*Ω*_ of the vanishing-gradient regularization term were kept constant for all networks. We also successfully trained networks with values for *G* and *λ*_*Ω*_ that were larger than the default values given in Table 1. When one or two parameters were modified to illustrate a particular training procedure, they are noted in the task descriptions. For instance, the number of trials used for each parameter update (gradient batch size) was the same in all networks except for the context-dependent integration task (to account for the large number of conditions) and sequence execution task (because of online training, where the number of trials is one). As a simple safeguard against extreme fine-tuning, we removed all weights below a threshold, *w*_min_, after training. We also note that, unlike in previous work (e.g., [5]), we used the same level of stimulus and noise for both training and testing.

**Table 1:** Parameters for stochastic gradient descent (SGD) training of recurrent neural networks (RNNs). Unless noted otherwise in the task description, networks were trained and run with the parameters listed here.

Parameter	Symbol	Default value
Learning rate	*η*	0.01
Maximum gradient norm	*G*	1
Multiplier for vanishing-gradient regularization *Ω*	*λ*_*Ω*_	2
Unit time constant	*τ*	100 ms
Time step (training)	Δ*t*	*τ*/5
Time step (testing)	Δ*t*	0.5 ms
Initial spectral radius of recurrent weight matrix	*ρ*	1.5
Gradient minibatch size	*N*_trials_	20
Baseline input	*u*^0^	0.2
Standard deviation for input noise	*σ*_in_	0.01
Standard deviation for recurrent noise	*σ*_rec_	0.15
Minimum weight threshold after training	*w*_min_	10^−4^

Code for generating the figures in this work are available from https://github.com/xjwanglab/pycog. The distribution includes code for training the networks, running trials, performing analyses, and creating the figures.

## Results

In this section we present the results of applying the training framework to well-known experimental paradigms in systems neuroscience: perceptual decision-making [61–63], context-dependent integration [5], multisensory integration [64], parametric working memory [34, 65], and eye-movement sequence generation [66]. In addition to establishing the relative ease of obtaining networks that perform the selected tasks, we show several single-neuron and population analyses associated with each paradigm. These analyses demonstrate that trained networks exhibit many, though not yet all, features observed in recorded neurons, and the study of these networks therefore has the potential to yield insights into biological neural circuits. A summary of the tasks can be found in Table 2.

**Table 2:** Summary of tasks. In the multisensory integration and parametric working memory tasks, networks receive both positively (pos.; increasing function) and negatively (neg.; decreasing function) tuned versions of the same input.

Fig	Task	Network inputs	Outputs	Features
Fig 2	Perceptual decision making (variable stimulus duration, VS; reaction-time, RT) [61–63]	Motion 1 Motion 2 Start of stimulus	Choice 1 Choice 2	Psychometric curves (VS, RT) Percent correct as a function of stimulus duration (VS) Reaction-time as a function of coherence, distribution (RT) Coherence-dependent firing rates (VS) Convergence of firing rates aligned to decision time (RT)
Fig 3	Perceptual decision making (fixed stimulus duration)	Motion 1 Motion 2	Choice 1 Choice 2	Psychometric curves No Dale’s principle vs. Dale’s principle Dense vs. constrained initial connectivity
Fig 4	Context-dependent integration [5]	Motion 1 Motion 2 Color 1 Color 2 Motion context Color context	Choice 1 Choice 2	Psychometric curves, gating State-space analysis Mixed selectivity of single-unit responses Distribution of regression coefficients
Fig 5	Context-dependent integration	Same	Same	Two areas
Fig 6	Multisensory integration [64]	Pos. tuned visual Neg. tuned visual Pos. tuned auditory Neg. tuned auditory Start of stimulus	Choice high Choice low	Psychometric curves with multisensory enhancement Heterogeneous selectivity in single-unit responses
Fig 7	Parametric working memory [34, 65]	Pos. tuned frequency Neg. tuned frequency	Choice *f*_1_ > *f*_2_ Choice *f*_1_ < *f*_2_	Heterogeneous tuning Correlation of tuning across population Change of tuning during trial
Fig 8	Sequence execution [66]	Targets (9) Sequence (8)	Eye position (*x*, *y*)	Continuous trials Online learning State-space analysis: hierarchical decision making

The tasks presented in this section represent only a small sample of the diversity of tasks used in neuroscience. In addition, we have chosen—in most cases arbitrarily—a simple set of constraints that do not necessarily reflect the full biological reality. Nevertheless, our work provides the foundation for further exploration of the constraints, regularizations, and network architectures required to achieve the greatest correspondence between trained RNNs and biological neural networks.

### Perceptual decision-making task

Many experimental paradigms in neuroscience require subjects to integrate noisy sensory stimuli in order to choose between two actions (Fig 1). Here we present networks trained to perform two variants of perceptual decision-making inspired by the two common variants of the random dot motion discrimination task [61–63]. For both versions, the network has 100 units (80 excitatory and 20 inhibitory) and receives two noisy inputs, one indicating evidence for choice 1 and the other for choice 2, and must decide which is larger. Importantly, the network is not explicitly told to integrate—it is instead only required to “make a decision” following the onset of stimulus by holding a high value in the output corresponding to the higher input, and a low value in the other.

**Fig 2:**
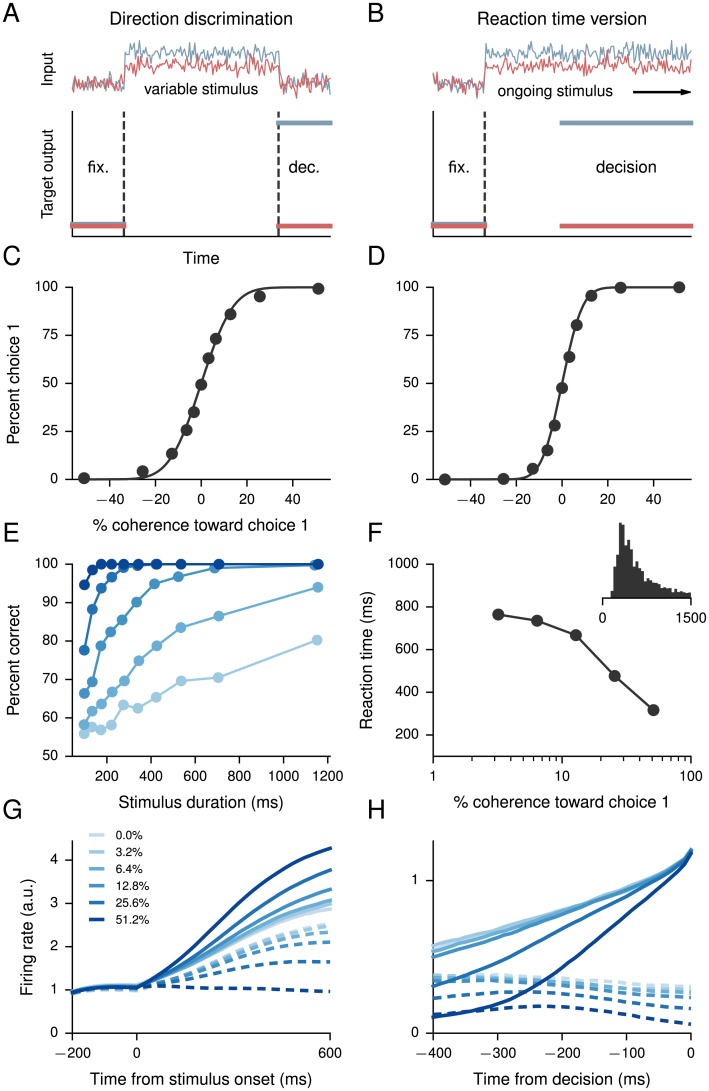
Perceptual decision-making task. (A) Inputs (upper) and target outputs (lower) for a perceptual decision-making task with variable stimulus duration, which we refer to as VS here. The choice 1 output must hold low during fixation (fix.), then high during the decision (dec.) period if the choice 1 input is larger than choice 2 input, low otherwise, and similarly for the choice 2 output. There are no constraints on output during the stimulus period. (B) Inputs and target outputs for the reaction-time version of the integration task, which we refer to as RT. Here the outputs are encouraged to respond after a short delay following the onset of stimulus. The reaction time is defined as the time it takes for the outputs to reach a threshold. (C) Psychometric function for the VS version, showing the percentage of trials on which the network chose choice 1 as a function of the signed coherence. Coherence is a measure of the difference between evidence for choice 1 and evidence for choice 2, and positive coherence indicates evidence for choice 1 and negative for choice 2. Solid line is a fit to a cumulative Gaussian distribution. (D) Psychometric function for the RT version. (E) Percentage of correct responses as a function of stimulus duration in the VS version, for each nonzero coherence level. (F) Reaction time for correct trials in the RT version as a function of coherence. Inset: Distribution of reaction times on correct trials. (G) Example activity of a single unit in the VS version across all correct trials, averaged within conditions after aligning to the onset of the stimulus. Solid (dashed) lines denote positive (negative) coherence. (H) Example activity of a single unit in the RT version, averaged within conditions and across all correct trials aligned to the reaction time.

In the variable stimulus-duration version of the task (Fig 2A), stimulus durations are drawn randomly from a truncated exponential distribution (we note that this is often called the “fixed-duration” version because the experimentalist sets the reaction time, in contrast to the “reaction-time” version in which the subject chooses when to respond). This minimizes the network’s ability to anticipate the end of the stimulus and therefore encourages the network to continue integrating information as long as the stimulus is present [63]. In the reaction-time version (Fig 2B), the network must respond soon after the onset of an ongoing stimulus. To control the speed-accuracy tradeoff, the target outputs during training did not require the network to commit to a decision immediately but instead after a short delay [62]; the delay determines the cost incurred for answering early but incorrectly versus correctly but at a later time.

All trials begin with a “fixation” period during which both outputs must maintain a low value, requiring the network to react only to the stimulus. The fixation can be enforced during training in several ways, including a variable fixation period whose duration is drawn from another truncated exponential distribution, or by introducing “catch trials” when no stimuli are presented. For simplicity, here we used a small proportion of catch trials mixed into the training, together with an additional, unambiguous start cue that signals the onset of stimulus.

Networks trained for both versions of the task show comparable performance in their psychometric functions (Fig 2C and 2D), which are the percentage of trials on which the network chose choice 1 as a function of the signed coherence. Coherence is a measure of the difference between evidence for choice 1 and evidence for choice 2, and positive coherence indicates evidence for choice 1 and negative for choice 2. In experiments with monkeys the signs correspond to inside and outside, respectively, the receptive field of the recorded neuron; although we do not show it here, this can be explicitly modeled by combining the present task with the model of “eye position” used in the sequence execution task (below). We emphasize that, unlike in the usual machine learning setting, our objective is not to achieve “perfect” performance. Instead, the networks were trained to an overall performance level of approximately 85% across all nonzero coherences to match the smooth psychometric profiles observed in behaving monkeys. We note that this implies that some networks exhibit a slight bias toward choice 1 or choice 2, as is the case with animal subjects unless care is taken to eliminate the bias through adjustment of the stimuli. Together with the input noise, the recurrent noise enables the network to smoothly interpolate between low-coherence choice 1 and low-coherence choice 2 trials, so that the network chooses choice 1 on approximately half the zero-coherence trials when there is no mean difference between the two inputs. Recurrent noise also forces the network to learn more robust solutions than would be the case without.

For the variable stimulus duration version of the decision-making task, we computed the percentage of correct responses as a function of the stimulus duration for different coherences (Fig 2E), showing that for easy, high-coherence trials the duration of the stimulus period only weakly affects performance [63]. In contrast, for difficult, low-coherence trials the network can improve its performance by integrating for a longer period of time. Fig 2G shows the activity of an example unit (selective for choice 1) across all correct trials, averaged within conditions after aligning to the onset of the stimulus. The activity shows a clear tuning of the unit to different signed coherences.

For the reaction-time version of the task, we defined a threshold for the output (here arbitrarily taken to be 1, slightly less than the target of 1.2 during training) that constituted a “decision.” The time it takes to reach this threshold is called the *reaction time*, and Fig 2F shows this reaction time as a function of coherence for correct trials, while the inset shows the distribution of reaction times on correct trials. In the case of the reaction-time version of the task, it is interesting to consider the activity of single units aligned to the decision time in each trial, which shows that the firing rate of the unit converges to a similar value for all positive coherences (Fig 2H) [62]. This is a nontrivial observation in both experiment [62] and model, as the decision threshold is only imposed on the outputs and not on the recurrent units themselves.

**Fig 3:**
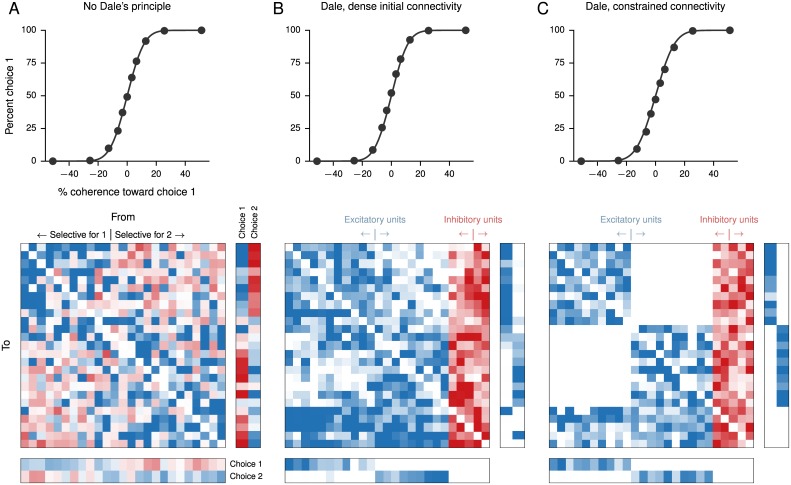
Perceptual decision-making networks with different constraints. (A) Psychometric function (percent choice 1 as a function of signed coherence) and connection weights (input, upper-right; recurrent, upper-left; and output, lower) for a network in which all weights may be positive or negative, trained for a perceptual decision-making task. Connections go from columns (“pre-synaptic”) to rows (“post-synaptic”), with blue representing positive weights and red negative weights. Different color scales (arbitrary units) were used for the input, recurrent, and output matrices but are consistent across the three networks shown. In the psychometric function, solid lines are fits to a cumulative Gaussian distribution. In this and the networks in *B* and *C*, self-connections were not allowed. In each case 100 units were trained, but only the 25 units with the largest absolute selectivity index (Eq 30) are shown, ordered from most selective for choice 1 (large positive) to most selective for choice 2 (large negative). (B) A network trained for the same task as in *A* but with the constraint that excitatory units may only project positive weights and inhibitory units may only project negative weights. All input weights were constrained to be excitatory, and the readout weights, considered to be “long-range,” were nonzero only for excitatory units. All connections except self-connections were allowed, but training resulted in a strongly clustered pattern of connectivity. Units are again sorted by selectivity but separately for excitatory and inhibitory units (20 excitatory, 5 inhibitory). (C) Same as *B* but with the additional constraint that excitatory recurrent units receiving input for choice 1 and excitatory recurrent units receiving input for choice 2 do not project to one another, and each group sends output to the corresponding choice.

To illustrate the effect of constraints on connectivity structure—but not on performance—we also trained three networks for the fixed stimulus-duration version of the task shown in Fig 2A. For these networks we did not use a start cue. In the first network, no constraints were imposed on the connection weights except for the absence of self-connections (Fig 3A). The second network was required to satisfy Dale’s principle, with a 4-to-1 ratio of the number of excitatory to inhibitory units, and purely excitatory inputs and outputs (Fig 3B). The third network was similar, but with the additional constraint that the inputs that signal evidence for choice 1 and choice 2 project to distinct groups of recurrent units and decisions are read out from the same group of excitatory units (Fig 3C). The two groups of excitatory units send zero excitatory projections to each other, communicating instead only through the inhibitory units and excitatory units that receive no inputs.

In all three cases, a clear structure could be discerned in the connectivity of the trained network by sorting the units by their selectivity index

$$d^{\prime }=\frac{\mu_{1}-\mu_{2}}{\sqrt{(\sigma_{1}^{2}+\sigma_{2}^{2})/2}},$$ (30)

where $\mu_{1},\sigma_{1}^{2}$ are the mean and variance of the unit’s activity, during the stimulus period, on trials in which the network chose choice 1, and similarly for $\mu_{2},\sigma_{2}^{2}$ for choice 2. For the network without separate excitatory and inhibitory units (Fig 3A), clustering manifests in the form of strong excitation among units with similar *d*′ and strong inhibition between units with different *d*′. The learned input weights also excite one population and inhibit the other. In the case of the network with separate excitatory and inhibitory populations (Fig 3B), units with different *d*′ interact primarily through inhibitory units [67]. Importantly, despite the fact that the recurrent weight matrix was initialized with dense, all-to-all connectivity, the two populations send fewer excitatory projections to each other after training. Similarly, despite the fact that the input weights initially send evidence for both choices to the two populations, after training the two groups receive evidence for different choices. Output weights also became segregated after training. In the third network this structure was imposed from the start, confirming that such a network could learn to perform the task (Fig 3C).

### Context-dependent integration task

In this section and the next we show networks trained for experimental paradigms in which making a correct decision requires integrating two separate sources of information. We first present a task inspired by the context-dependent integration task of [5], in which a “context” cue indicates that one type of stimulus (the motion or color of the presented dots) should be integrated and the other completely ignored to make the optimal decision.

**Fig 4:**
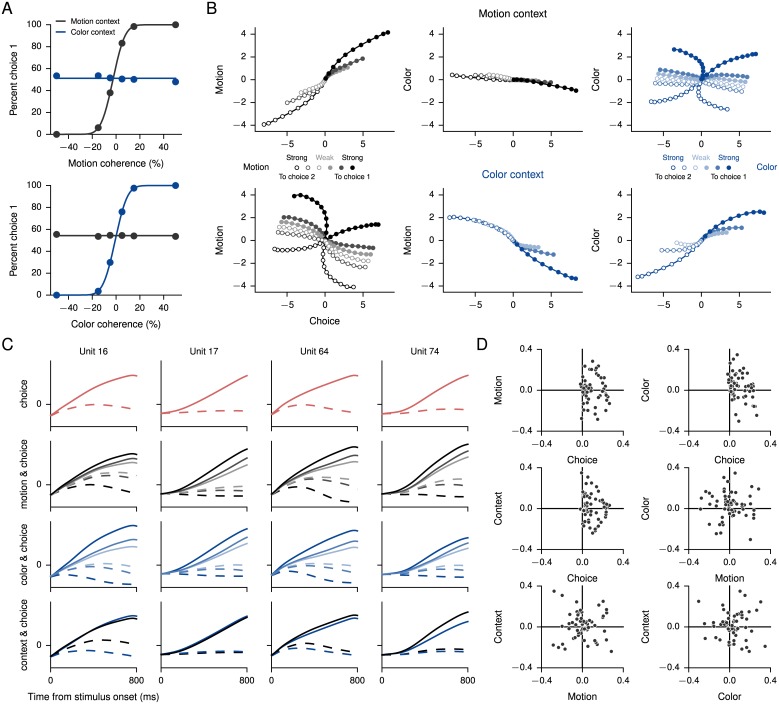
Context-dependent integration task. (A) Psychometric function, showing the percentage of trials on which the network chose choice 1 as a function of the signed motion (upper) and signed color (lower) coherence in motion-context (black) and color-context (blue) trials. (B) Average population responses in state space during the stimulus period, projected to the 3-dimensional subspace capturing variance due to choice, motion, and color as in [5]. Only correct trials were included. The task-related axes were obtained through a linear regression analysis. Note that “choice” here has a unit-specific meaning that depends on the preferred choice of the unit as determined by the selectivity index (Eq 30). For both motion (black) and color (blue), coherences increase from light to dark. Upper plots show trials during the motion context, and lower plots show trials during the color context. (C) Normalized responses of four recurrent units during the stimulus period show mixed representation of task variables. Solid lines indicate the preferred choice and dashed lines the nonpreferred choice of each unit. (D) Denoised regression coefficients from the linear regression analysis. By definition, the coefficients for choice are almost exclusively positive.

A network trained for the context-dependent integration task is able to integrate the relevant input while ignoring the irrelevant input. This is reflected in the psychometric functions, the percentage of trials on which the network chose choice 1 as a function of the signed motion and color coherences (Fig 4A). The network contains a total of 150 units, 120 of which are excitatory and 30 inhibitory. The training protocol was very similar to the (fixed-duration) single-stimulus decision-making task except for the presence of two independent stimuli and a set of context inputs that indicate the relevant stimulus. Because of the large number of conditions, we increased the number of trials for each gradient update to 50.

Previously, population responses in the monkey prefrontal cortex were studied by representing them as trajectories in neural state space [5]. This was done by using linear regression to define the four orthogonal, task-related axes of choice, motion, color, and context. The projection of the population responses onto these axes reveals how the different task variables are reflected in the neural activity. Fig 4B shows the results of repeating this analysis [5] with the trained network during the stimulus period. The regression coefficients (Fig 4D) reveal additional relationships between the task variables, which in turn reflect the mixed selectivity of individual units to different task parameters as shown by sorting and averaging trials according to different criteria (Fig 4C).

**Fig 5:**
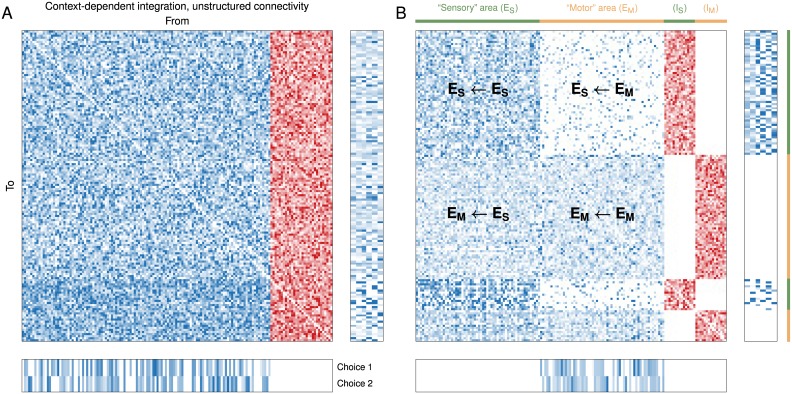
Constraining the connectivity. Connectivity after training for the context-dependent integration task (Fig 4), when the connection matrix is (A) unstructured and (B) structured. Both networks consist of 150 units (120 excitatory, 30 inhibitory). In *B* the units are divided into two equal-sized “areas,” each with a local population of inhibitory units (*I*_*S*_ and *I*_*M*_) that only project to units in the same area. The “sensory” area (green) receives excitatory inputs and sends dense, “long-range” excitatory feedforward connections *E*_*M*_ ← *E*_*S*_ to the “motor” area (orange) from which the outputs are read out. The sensory area receives sparse excitatory feedback projections *E*_*S*_ ← *E*_*M*_ from the motor area.

As a proof of principle, we trained an additional network that could perform the same task but consisted of separate “areas,” with one area receiving inputs and the other sending outputs (Fig 5B), which can be compared to the unstructured connectivity of the original network (Fig 5A). Here each area is conceived of as a cortical area containing a group of inhibitory units that only project locally to excitatory and inhibitory units in the same area. Thus there are no interareal connections originating from inhibitory units. The “sensory” area that receives inputs sends dense, “long-range” excitatory feedforward connections to the “motor” area from which outputs are read out, and receives “sparse” (connection probability 0.2) excitatory feedback projections from the motor area. This example illustrates the promise of using RNNs to explore how large-scale function may arise in the brain.

### Multisensory integration task

**Fig 6:**
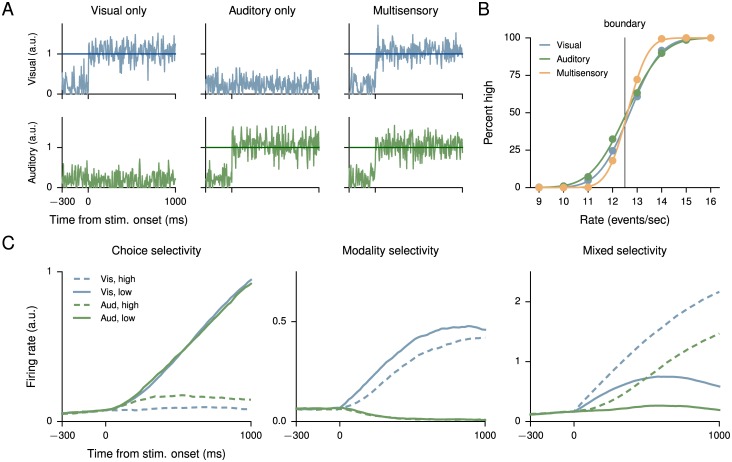
Multisensory integration task. (A) Example inputs for visual only (left), auditory only (middle), and multisensory (both visual and auditory, right) trials. Network units receive both positively tuned (increasing function of event rate) and negatively tuned (decreasing function of event rate) inputs; panels here show positively tuned input corresponding to a rate of 13 events/sec, just above the discrimination boundary. As in the single-stimulus perceptual decision-making task, the outputs of the network were required to hold low during “fixation” (before stimulus onset), then the output corresponding to a high rate was required to hold high if the input was above the decision boundary and low otherwise, and vice versa for the output corresponding to a low rate. (B) Psychometric functions (percentage of choice high as a function of the event rate) for visual, auditory, and multisensory trials show multisensory enhancement. (C) Sorted activity on visual only and auditory only trials for three units selective for choice (high vs. low, left), modality (visual vs. auditory, middle), and both (right).

The multisensory integration task of [64] also presents the animal—rats, in this case—with two sources of information. In contrast to the previous task, however, in the multisensory integration task it is advantageous for the animal to integrate both sources of information when they are available. Specifically, visual flashes and auditory clicks were presented at rates between 9 events/sec and 16 events/sec, and the animal was required to determine whether the inputs were below or above the threshold of 12.5 events/sec. When both visual and auditory inputs were present, they were congruent (presented at the same rate). A network trained for this task is also given one or more congruent inputs, and can improve its performance by combining both inputs when they are available (Fig 6A and 6B). The network contains 150 units, 120 of which are excitatory and 30 inhibitory. A third of the units in the network (both excitatory and inhibitory) received only visual input, another third only auditory input, and the remaining third did not receive any input. Outputs were read out from the entire excitatory population.

The training was again mostly similar to the (fixed-duration) single-stimulus perceptual decision-making task, except for the presence of two congruent inputs on multisensory trials. However, in the present task the network must determine whether the given input is larger or smaller than an arbitrary strength, in contrast to the previous integration tasks where two inputs are compared to each other. As a result, giving the network both positively tuned (increasing function of event rate) and negatively tuned (decreasing function of event rate) inputs [68] greatly improved training. Although gradient-descent training can find a solution when the inputs are purely positively tuned, this results in much longer training times and more idiosyncratic unit activities. This illustrates that, while RNN training methods are powerful, they must be supplemented with knowledge gained from experiments and previous modeling studies. As in experimentally recorded neurons, the units of the network exhibit heterogeneous responses, with some units showing selectivity to choice, others to modality, and still others showing mixed selectivity (Fig 6C).

The context-dependent and multisensory integration tasks represent the two end-cases of when two separate sources of information are available for making a decision. It is of great interest for future inquiry how the *same* network or set of networks may switch from completely ignoring one input to using both inputs to make the optimal decision depending on the task.

### Parametric working memory task

**Fig 7:**
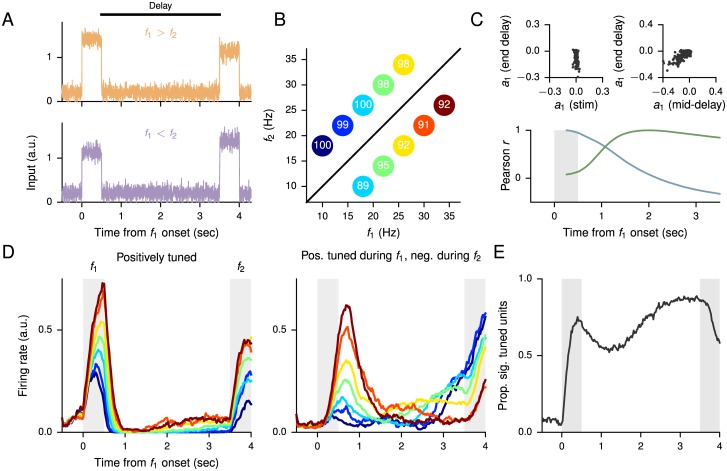
Parametric working memory task. (A) Sample positively tuned inputs, showing the case where *f*_1_ > *f*_2_ (upper) and *f*_1_ < *f*_2_ (lower). Recurrent units also receive corresponding negatively tuned inputs. (B) Percentage of correct responses for different combinations of *f*_1_ and *f*_2_. This plot also defines the colors used for each condition, labeled by *f*_1_, in the remainder of the figure. Due to the overlap in the values of *f*_1_, there are 7 distinct colors representing 10 trial conditions. (C) Lower: Correlation of the tuning *a*_1_ (see text) at different time points to the tuning in the middle of the first stimulus period (blue) and middle of the delay period (green). Upper: The tuning at the end of delay vs. middle of the first stimulus (left) and the end of delay vs. middle of the delay (right). (D) Single-unit activity for a unit that is positively tuned for *f*_1_ during both stimulus periods (left), and for a unit that is positively tuned during the first stimulus period but negatively tuned during the second stimulus period (right). (E) Proportion of significantly tuned units based on a simple linear regression of the firing rates as a function of *f*_1_ at each time point.

One of the most important—and therefore one of the most widely studied—cognitive functions is working memory, the ability to maintain and manipulate information for several seconds during the planning and execution of a task [69, 70]. Working memory has notably been studied in the context of both oculomotor parametric working memory [71] and vibrotactile frequency discrimination [1, 65], and here we trained a network to perform a task based on the frequency discrimination task. In this task, two temporally separated stimuli, represented by constant inputs whose magnitudes are proportional to the frequency (Fig 7A), are presented and the network must determine which of the two is of higher frequency. This requires the network to remember the frequency of the first input *f*_1_ throughout the 3-second delay period in order to compare to the second input *f*_2_ at the end of the delay period. The network contains a total of 500 units (400 excitatory, 100 inhibitory), with a connection probability of 0.1 from excitatory units to all other units and 0.5 from inhibitory units to all other units; these connection probabilities are consistent with what is known for local microcircuits in cortex [52, 53]. During training only, the delay was varied by uniformly sampling from the range 2.5–3.5 seconds. As in the multisensory integration task, because the network must compare a single input against itself (rather than comparing two simultaneous inputs to each other), it is helpful for the network to receive both positively tuned and negatively tuned inputs.

The network’s performance on each condition is shown in Fig 7B. Based on the experimental results, we trained the network until the lowest percentage of correct responses in any condition exceeded 85%; for most conditions the performance is much higher [34]. Several different types of behavior are observed in the unit activities. For instance, some units are positively tuned for the frequency *f*_1_ during both stimulus periods (Fig 7D, left). Other units are positively tuned for *f*_1_ during the first stimulus period but negatively tuned during the second (Fig 7D, right); the switch can occur at various times during the delay. Following [34], we performed a simple linear analysis of the tuning properties of units at different times by fitting the firing rate at each time point to the form *r*(*t*) = *a*_0_(*t*) + *a*_1_(*t*)*f*_1_. The results are presented in Fig 7C, which shows the correlation of *a*_1_ between different time points across the population, and Fig 7E, which shows the percentage of significantly tuned (two-sided *p*-value <0.05) units at different times. The latter shows trends similar to those observed in monkeys.

### Eye-movement sequence execution task

**Fig 8:**
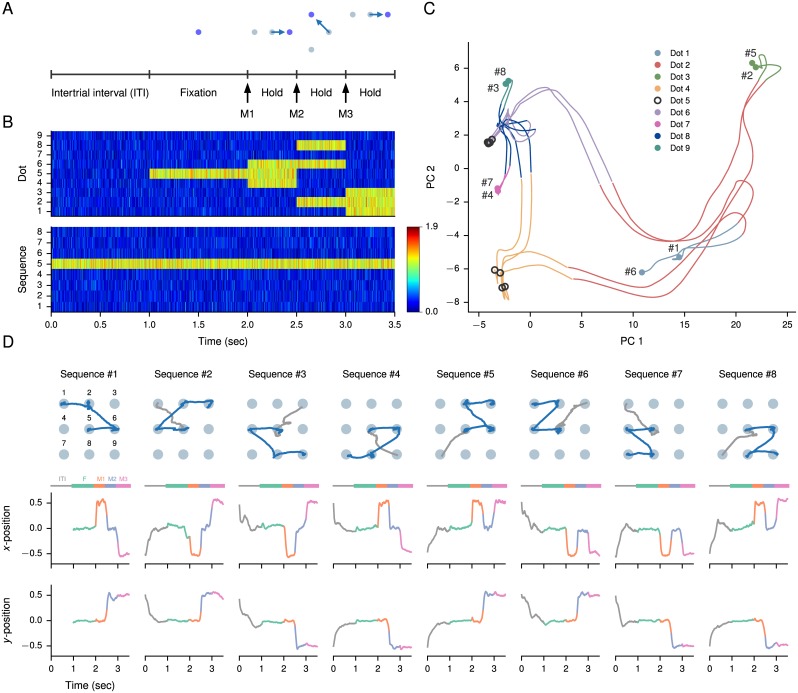
Eye-movement sequence execution task. (A) Task structure (for Sequence 5) and (B) sample inputs to the network. During the intertrial interval (ITI) the network receives only the input indicating the current sequence to be executed. Fixation is indicated by the presence of a fixation input, which is (the central) one of 9 possible dot positions on the screen. During each movement, the current dot plus two possible target dots appear. (C) State-space trajectories during the three movements M1, M2, and M3 for each sequence, projected on the first two principal components (PCs) (71% variance explained, note the different axis scales). The network was run with zero noise to obtain the plotted trajectories. The hierarchical organization of the sequence of movements is reflected in the splitting off of state-space trajectories. Note that all sequences start at fixation, or dot 5 (black), and are clustered here into two groups depending on the first move in the sequence. (D) Example run in which the network continuously executes each of the 8 sequences once in a particular order; the network can execute the sequences in any order. Each sequence is separated by a 1-second ITI during which the eye position returns from the final dot in the previous trial to the central fixation dot. Upper: Eye position in “screen” coordinates. Lower: *x* and *y*-positions of the network’s outputs indicating a point on the screen. Note the continuity of dynamics across trials.

An experimental paradigm that is qualitatively very different from the previous examples involves the memorized execution of a sequence of motor movements, and is inspired by the task of [66]. An important difference from a modeling point of view in this case is that, unlike in previous tasks where we interpreted the outputs as representing a decision variable between two choices, here we interpret the network’s two outputs to be the *x* and *y*-coordinates corresponding to the monkey’s eye position on the screen. After maintaining fixation on the central dot for 1 second, the task is to execute a sequence of three eye movements and hold for 500 ms each (Fig 8A). For each movement, two targets are presented as inputs to indicate the possible moves in addition to the current dot; although the targets could be presented in a more realistic manner—in a tuning curve-representation, for instance—here we use the simple encoding in which each input corresponds to a potential target location. Throughout the trial, an additional input is given that indicates which sequence, out of a total of 8, is being executed (Fig 8B).

For this task we trained a 200-unit (160 excitatory, 40 inhibitory) network on a trial-by-trial basis, i.e., the network parameters were updated after each trial. This corresponds to setting the gradient minibatch size to 1. Moreover, the network was run “continuously,” without resetting the initial conditions for each trial (Fig 8D). During the intertrial interval (ITI), the network returns its eye position to the central fixation point from its location at the end of the third movement, so that the eye position is in the correct position for the start of the next fixation period. This occurs even though the target outputs given to the network during training did not specify the behavior of the outputs during the ITI, which is interesting for future investigation of such networks’ ability to learn tasks with minimal supervision.

During training, each sequence appeared once in a block of 8 randomly permuted trials. Here we used a time constant of *τ* = 50 ms to allow faster transitions between dots. For this task only, we used a smaller recurrent noise of *σ*_rec_ = 0.01 because the output values were required to be more precise than in previous tasks, and did not limit readout to excitatory units to allow for negative coordinates. We note that, in the original task of [66] the monkey was also required to infer the sequence it had to execute in a block of trials, but we did not implement this aspect of the task. Instead, the sequence was explicitly indicated by a separate set of inputs.

Because the sequence of movements are organized hierarchically—for instance, the first movement must decide between going left and going right, the next movement must decide between going up and going down, and so forth—we expect a hierarchical trajectory in state space. This is confirmed by performing a principal components analysis and projecting the network’s dynamics onto the first two principal components (PCs) computed across all conditions (Fig 8C).

## Discussion

In this work we have described a framework for gradient descent-based training of excitatory-inhibitory RNNs, and demonstrated the application of this framework to tasks inspired by well-known experimental paradigms in systems neuroscience.

Unlike in machine learning applications, our aim in training RNNs is not simply to maximize the network’s performance, but to train networks so that their performance matches that of behaving animals while both network activity and architecture are as close to biology as possible. We have therefore placed great emphasis on the ability to easily explore different sets of constraints and regularizations, focusing in particular on “hard” constraints informed by biology. The incorporation of separate excitatory and inhibitory populations and the ability to constrain their connectivity is an important step in this direction, and is the main contribution of this work.

The framework described in this work for training RNNs differs from previous studies [5, 8] in several other ways. In this work we use threshold (rectified) linear units for the activation function of the units. Biological neurons rarely operate in the saturated firing-rate regime, and the use of an unbounded nonlinearity obviates the need for regularization terms that prevent units from saturating [8]. Despite the absence of an upper bound, all firing rates nevertheless remained within a reasonable range. We also favor first-order SGD optimization over second-order HF methods. This is partly because of SGD’s widely acknowledged effectiveness in current approaches to machine learning, but also because gradient descent, unlike HF, allows for trial-by-trial learning and may ultimately be more easily related to synaptic learning rules in the brain [39, 40].

Eqs 1–3 are a special case of the more general set of equations for RNNs given in S1 Text, which in turn represent only one of many possible RNN architectures. For instance, machine learning applications typically employ a type of RNN known as Long Short-Term Memory (LSTM), which uses multiplicative gates to facilitate learning of long-term dependencies and currently represents one of the most powerful methods for solving sequence-related problems [72]. For reasons of biological interpretation, in our implementation we only consider generalizations that retain the “traditional” RNN architecture given by Eqs 1–3. These generalizations include additive bias terms in recurrent and output units (corresponding to variable thresholds), different time constants for each unit (e.g., faster inhibitory units), correlated noise [73], and other types of nonlinearities besides simple rectification (e.g., supralinear [74] or saturating *f-I* curves) for either recurrent units or outputs. We found that biases, though not used for the networks in this work, can improve training in some situations by endowing the network with greater flexibility. The choice of output nonlinearity can be particularly relevant when considering the precise meaning of the outputs, such as whether the outputs are considered a decision variable, probability distribution, or eye position. Probability output models are useful, for instance, when the animal’s confidence about its decision is of interest in addition to its actual decision.

**Fig 9:**
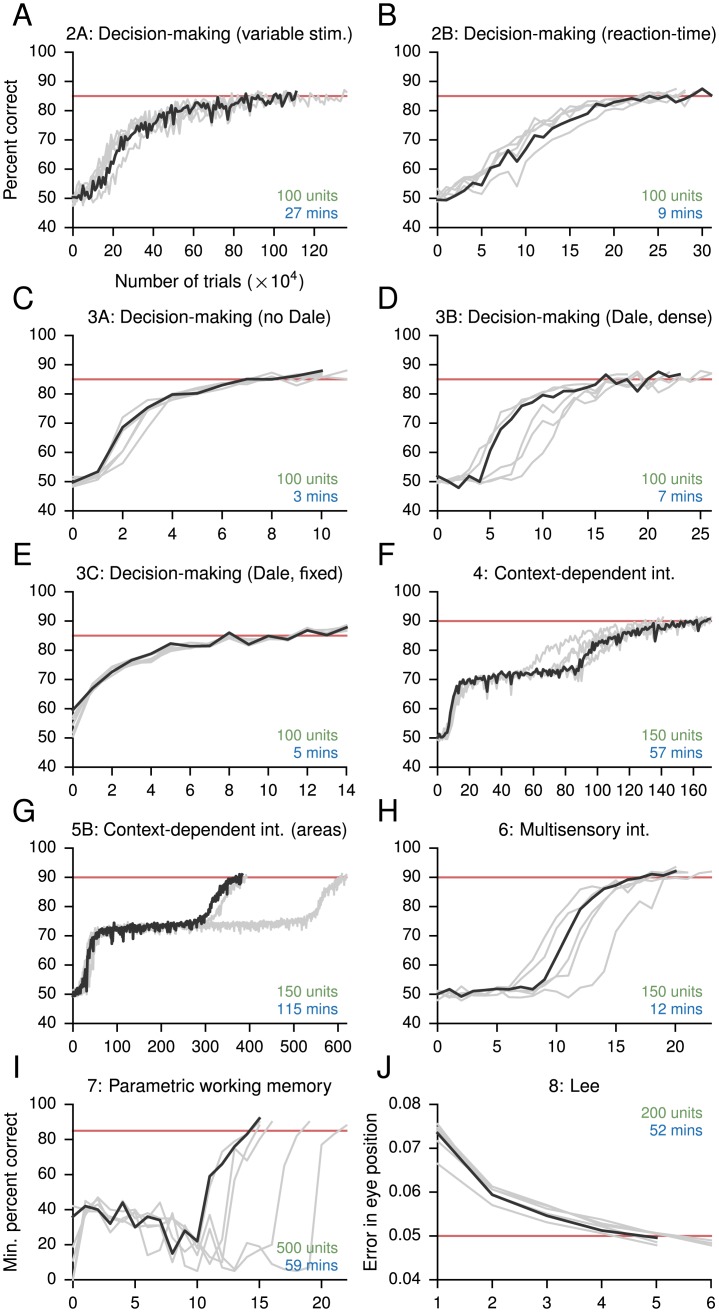
Estimated performance during training for networks in the Results. (A)-(I) Percentage of correct responses. (J) Error in eye position. For each network the relevant figure in the main text and a brief description are given. Black lines are for the networks shown in the main text, while gray lines show the performance for 5 additional networks trained for the same tasks but using different initial weights. Red lines indicate the target performance; training terminated when the mean performance on several (usually 5) evaluations of the validation dataset exceeded the target performance. In *I* the target performance indicates the minimum, rather than mean, percentage of correct responses across conditions. The number of recurrent units (green) is indicated for each network. The number of minutes (in “real-time”) needed for training (blue) are estimates for a MacBook Pro running OS X Yosemite 10.10.4, with a 2.8 GHz Intel Core i7 CPU and 16 GB 1600 MHz DDR3 memory. GPUs were not used in the training of these networks.

Several works [5, 7, 34] have now demonstrated the value of trained RNNs in revealing circuit mechanisms embedded in large neural populations. In addition to the pioneering work on uncovering a previously unknown selection mechanism for context-dependent integration of sensory inputs in [5], work reported in [7] used trained RNNs to reveal possible dynamical implementations of response criterion modulation in a perceptual detection task under temporal uncertainty. Yet, more recent methods for training networks have not been widely available or easily accessible to the neuroscience community. We have endeavored to change this by providing an easy-to-use but flexible implementation of our framework that facilitates further modifications and extensions. For the tasks featured in this work, the amount of time needed for training was relatively short and largely consistent across different initializations (Fig 9), and could be made even shorter for exploratory training by reducing the network size and noise level. Although further improvements can be made, our results already demonstrate that exploratory network training can be a practical and useful tool for neuroscientists. Moreover, while the present learning rule is not biologically plausible, it is of interest whether the behavioral trajectory of learning can be made similar to that of animals learning the same tasks. In particular, the question of how many trials are needed to learn a given task in model RNNs and animals merits further investigation.

Many interesting and challenging questions remain. Although RNNs of rate units often provide a valuable starting point for investigating both the dynamical and neural computational mechanisms underlying cognitive functions, they will not always be the most appropriate level of description for biological neural circuits. In this work we have not addressed the question of how the firing rate description given by RNN training can be properly mapped to the more realistic case of spiking neurons, and indeed it is not completely clear, at present, how spiking neurons may be directly trained for general tasks using this type of approach. In this work we have only addressed tasks that could be formulated in the language of supervised learning, i.e., the correct outputs were explicitly given for each set of inputs. Combining RNN training with reinforcement learning methods [75–77] will be essential to bringing network training closer to the reward-based manner in which animals are trained. Despite limitations, particularly on the range of tasks that can be learned, progress on training spiking neurons with STDP-type rules and reinforcement learning is promising [78–80], and future work will incorporate such advances. Other physiologically relevant phenomena such as bursting, adaptation, and oscillations are currently not captured by our framework, but can be incorporated in the future; adaptation, for example, can be included in phenomenological form appropriate to a rate model [81, 82].

We have also not addressed what computational advantages are conferred, for example, by the existence of separate excitatory and inhibitory populations, instead taking it as a biological fact that must be included in models of animal cognition. Indeed, although our discussion has focused on the distinction between excitatory and inhibitory neurons, the functional role of inhibitory units may only become apparent when the full diversity of excitatory and inhibitory neuronal morphology and physiology, their layer and type-specific distribution and connectivity [49, 50], and domain-specific (e.g., dendritic versus somatic) targeting of excitatory pyramidal cells by interneurons [23, 25, 27, 51] in the brain are taken into account. Some of these phenomena can already be implemented in the framework by fixing the pattern of connectivity between groups of units (corresponding, for example, to different layers in a cortical column), and future work will explore the implications of such structure on network dynamics.

Finally, although we have performed a few basic analyses of the resulting networks, we have not addressed the detailed mechanisms by which networks accomplish their tasks. In this regard, although state-space analyses of fixed and “slow” points [83] are illuminating they do not yet explain how the network’s connectivity, combined with the nonlinear activation functions, lead to the observed neural trajectories. Discovering general methods for the systematic analysis of trained networks remains one of the most important areas of inquiry if RNNs are to provide useful insights into the operation of biological neural circuits. As a platform for theoretical investigation, trained RNNs offer a unified setting in which diverse cognitive computations and mechanisms can be studied. Our results provide a valuable foundation for tackling this challenge by facilitating the generation of candidate networks to study, and represent a fruitful interaction between modern machine learning and neuroscience.

## Supporting Information

S1 Code Example task specification: perceptual decision-making task. (PY)

S1 Text General equations for a recurrent neural network. (PDF)
