# Supplementary material for: Training Excitatory-Inhibitory Recurrent Neural Networks for Cognitive Tasks: A Simple and Flexible Framework
Source: PLoS Comput Biol. 2016 Feb 29;12(2):e1004792. doi: 10.1371/journal.pcbi.1004792 (PMC4771709; doi:10.1371/journal.pcbi.1004792)
Supplement: S1 Text — (PDF) [file pcbi.1004792.s002.pdf]

S1 Text: General equations for a recurrent neural network.

The networks presented in the main text were described by Eqs 1-3, which constitute a special case of the more general equations

$$\boldsymbol{\tau} \odot \dot{\mathbf{x}} = -\mathbf{x} + W^{\text{rec}}\mathbf{r} + \mathbf{b}^{\text{rec}} + W^{\text{in}}\mathbf{u} + \sqrt{2\boldsymbol{\tau}\sigma_{\text{rec}}^2} \odot \boldsymbol{\xi}, \tag{1}$$

$$\mathbf{r} = f(\mathbf{x}), \tag{2}$$

$$\mathbf{z} = g(W^{\text{out}}\mathbf{r} + \mathbf{b}^{\text{out}}), \tag{3}$$

where $\odot$ denotes element-wise multiplication of vectors; thus each unit is allowed to have a different time constant. The additional terms $\mathbf{b}^{\text{rec}}$ and $\mathbf{b}^{\text{out}}$ denote biases to the recurrent units and outputs, respectively. The nonlinear function $f(\mathbf{x})$ converts input currents into firing rates, while the nonlinear function $g(\mathbf{x})$ may be considered a more general mapping from the recurrent units to the output model (decision variable, probability distribution, eye position, etc.). Examples of point-wise nonlinearities for either f or g are the hyperbolic tangent $\tanh(x)$, sigmoid $1/(1+e^{-x})$, rectified linearity $[x]_+ = \max(0, x)$, rectified supralinearity $([x]_+)^n$ for $n > 1$ [74], and rectified hyperbolic tangent $\tanh\,[x]_+$. When the outputs are interpreted as a normalized probability distribution it is also natural to use the softmax function,

$$[g(\mathbf{y})]_\ell = \frac{\exp(y_\ell)}{\sum_{m=1}^{N_{\text{out}}} \exp(y_m)}. \tag{4}$$

Since the notion of excitatory and inhibitory neurons is most meaningful if firing rates are non-negative, and firing rates in cortex rarely saturate to their bounds, we used rectified linear units in the main text. Finally, the noise term $\boldsymbol{\xi}$ is not restricted to N independent Gaussian processes; instead, the entire distribution can be drawn from a multivariate normal distribution with an arbitrary covariance structure, thereby allowing us to study the effect of correlated noise in RNNs [73].

It is also desirable to choose appropriate measures for the difference between the actual network outputs $\mathbf{z}$ and target outputs $\mathbf{z}^{\text{target}}$ at each time point depending on the output nonlinearity. In the main text, we used the simplest pairing of a linear readout with sum-of-squares loss function. In the case where each output represents an independent probability we can use sigmoid outputs with the binary cross entropy (CE) loss

$$\mathcal{L}_{\text{binary-CE}} = -\sum_{\ell=1}^{N_{\text{out}}} \Big[z_\ell^{\text{target}} \log z_\ell + (1 - z_\ell^{\text{target}}) \log(1 - z_\ell) \Big]. \tag{5}$$

If all the outputs together represent one probability ("1-of-N" encoding) and therefore the softmax function of Eq 4 is used, then it is more appropriate to use the categorical CE loss

$$\mathcal{L}_{\text{categorical-CE}} = -\sum_{\ell=1}^{N_{\text{out}}} z_\ell^{\text{target}} \log z_\ell. \tag{6}$$
